# Interpretable machine learning models for predicting clinical pregnancies associated with surgical sperm retrieval from testes of different etiologies: a retrospective study

**DOI:** 10.1186/s12894-024-01537-1

**Published:** 2024-07-29

**Authors:** Shun-shun Cao, Xiao-ming Liu, Bo-tian Song, Yang-yang Hu

**Affiliations:** 1 Pediatric Endocrinology, Genetics and Metabolism, The Second Affiliated Hospital of Wenzhou Medical University, Wenzhou, Zhejiang 325000 China; 2 Reproductive Medicine Center, Obstetrics and Gynecology, The Second Affiliated Hospital of Wenzhou Medical University, Wenzhou, Zhejiang 325000 China

**Keywords:** Surgical sperm retrieval, Clinical pregnancy, Machine learning, SHapley Additive exPlanation

## Abstract

**Background:**

The relationship between surgical sperm retrieval of different etiologies and clinical pregnancy is unclear. We aimed to develop a robust and interpretable machine learning (ML) model for predicting clinical pregnancy using the SHapley Additive exPlanation (SHAP) association of surgical sperm retrieval from testes of different etiologies.

**Methods:**

A total of 345 infertile couples who underwent intracytoplasmic sperm injection (ICSI) treatment with surgical sperm retrieval due to different etiologies from February 2020 to March 2023 at the reproductive center were retrospectively analyzed. The six machine learning (ML) models were used to predict the clinical pregnancy of ICSI. After evaluating the performance characteristics of the six ML models, the Extreme Gradient Boosting model (XGBoost) was selected as the best model, and SHAP was utilized to interpret the XGBoost model for predicting clinical pregnancies and to reveal the decision-making process of the model.

**Results:**

Combining the area under the receiver operating characteristic curve (AUROC), accuracy, precision, recall, F1 score, brier score, and the area under the precision-recall (P-R) curve (AP), the XGBoost model has the best performance (AUROC: 0.858, 95% confidence interval (CI): 0.778–0.936, accuracy: 79.71%, brier score: 0.151). The global summary plot of SHAP values shows that the female age is the most important feature influencing the model output. The SHAP plot showed that younger age in females, bigger testicular volume (TV), non-tobacco use, higher anti-müllerian hormone (AMH), lower follicle-stimulating hormone (FSH) in females, lower FSH in males, the temporary ejaculatory disorders (TED) group, and not the non-obstructive azoospermia (NOA) group all resulted in an increased probability of clinical pregnancy.

**Conclusions:**

The XGBoost model predicts clinical pregnancies associated with testicular sperm retrieval of different etiologies with high accuracy, reliability, and robustness. It can provide clinical counseling decisions for patients with surgical sperm retrieval of various etiologies.

**Supplementary Information:**

The online version contains supplementary material available at 10.1186/s12894-024-01537-1.

## Introduction

The prevalence of infertility is gradually increasing worldwide, with about 8–12% of couples suffering from infertility and 10–20% of infertile men suffering from azoospermia [1, 2]. Decreased male fertility is often associated with testicular dysfunction, endocrine disruption, poor lifestyle, congenital developmental abnormalities, radiation, endocrine disruptor exposure, and aging, but 40% of male infertility is idiopathic and has a novel single gene linkage in the pathogenesis [3, 4]. With increasing infertility, the use of assisted reproductive technologies has risen dramatically in the last decade, but the success rate of intracytoplasmic sperm injection (ICSI) depends on several factors, and in particular, has a significant relationship with sperm quality [5]. Azoospermia is categorized into obstructive azoospermia (OA) and non-obstructive azoospermia (NOA), and sperm are often obtained for ICSI in in vitro fertilization (IVF) centers using testicular sperm aspiration (TESA) and microdissection testicular sperm extraction (mTESE) [6]. TESA is a more commonly used procedure for extracting male sperm in assisted reproduction techniques than mTESE.

TESA is commonly used in cases where the male partner is unable to ejaculate or has no available high-quality sperm on the day of oocyte retrieval, including erectile dysfunction (ED), temporary ejaculatory disorders (TED), complete retrograde ejaculation, OA, necrospermia, and high sperm deoxyribonucleic acid (DNA) fragmentation [7–9]. mTESE is most commonly used for testicular sperm retrieval in NOA. The uncertainty of treatment outcomes in assisted reproductive technology and the variation in the quality of testicular spermatozoa obtained by surgical sperm retrieval for different etiologies affect clinical pregnancy with ICSI treatment to varying degrees. Therefore, there is a need to develop a predictive model to assess and interpret the clinical pregnancy outcomes of ICSI for counseling infertile couples.

Machine learning (ML) is a type of artificial intelligence that overcomes the limits of expert systems by having manually written rules replaced by rules discovered manually from data, allowing ML systems to learn from data and explain unknown situations [10]. More traditional logistic regression models have been used to predict IVF outcomes by female factors [11, 12]. At the same time, fewer reports have been made on the prediction of clinical pregnancy in ICSI by male factors, especially the prediction model of clinical pregnancy in ICSI by the use of testicular spermatozoa of different etiologies has been reported for the first time. This study aimed to develop six ML models for the prediction of clinical pregnancy. We selected ML models with optimal performance and used ML visualization based on the SHapley Additive exPlanation (SHAP) to determine the contribution of surgical sperm retrieval for different etiologies in predicting clinical pregnancy [13].

## Materials and methods

### Data source and study design

This was a retrospective study. In this study, 420 infertile couples who underwent surgical testicular sperm retrieval with ICSI for different etiologies at the IVF center of the Second Affiliated Hospital of Wenzhou Medical University between February 2020 and March 2023 were selected, of which 345 cases met the inclusion criteria. The present study protocol was reviewed and approved by the Institutional Review Board of the Second Affiliated Hospital of Wenzhou Medical University (approval No. 2022-K-196-01). All participants signed written informed consent.

Inclusion criteria included: ⑴ female age ≤ 40 years old, ⑵ ICSI and embryo transfer in fresh cycles, ⑶ informed consent has been obtained, ⑷ TESA or mTESE was performed in the male on the day of female ovum retrieval, ⑸ The male partner was not given medication affecting sperm before TESA or mTESE, ⑹ the female partner is free from reproductive and systemic diseases that significantly affect clinical pregnancy in ICSI, ⑺ stimulation of ovulation using a uniform follicular length program, and ⑻ no fertility-related genetic or chromosomal abnormalities in either partner. Exclusion criteria included: ⑴ important data missing, ⑵ no complete fresh IVF treatment was performed, ⑶ other ICSI treatment options, and ⑷ sexually transmitted diseases and psychogenic ED.

### Groupings and definitions

According to the different etiologies, the spermatozoa obtained by surgical sperm extraction on the day of ovum retrieval were divided into 103 cases in the NOA group, 81 cases in the OA group, 95 cases in the ED group, and 66 cases in the TED group.

ED is defined according to the European Association of Urology guidelines as the persistent or recurrent inability to achieve or maintain a penile erection sufficiently satisfactory to satisfy sexual intercourse in response to appropriate sexual stimulation for at least 6 months [14]. The diagnosis of ED relies on medical history data, the international index of erectile function 5 (IIEF-5), physical examination, color Doppler ultrasonography, blood parameters, and history of previous medications, and the patients with ED in this study had predominantly organic erectile dysfunction [15]. Definition of azoospermia according to the 2021 World Health Organization laboratory manual for the examination and processing of human semen (6th edition) guidelines 3 analyses of semen were performed, and no sperm were observed at high magnification in at least 2 samples analyzed more than 2 weeks apart [16]. TESA can be used to identify OA and NOA, as well as mTESE is the gold standard for surgical sperm extraction in patients with NOA [17]. TED is a condition in which the patient can normally ejaculate through masturbation or sexual intercourse and fail to ejaculate on the day of female oocyte retrieval due to psycho-psychological factors. Females’ serum follicle-stimulating hormone (FSH) and anti-müllerian hormone (AMH) sampling and analysis were performed on days 2–3 of the menstrual period.

### TESA and mTESE operating procedure

The TESA surgical approach was similar to that described by authors Cito G et al. [18]. The operator was sterilized, the surgical towel was spread, the patient was anesthetized by infiltration of the scrotal skin to the testicular leucomembrane layer by layer using 5 ml of lidocaine, and the testis was slowly punctured using a sharp pointed puncture needle with a side hole attached to a 10 ml syringe, and the testicular tissue was obtained by negative pressure suction and quickly placed into a petri dish to be passed to the embryologists for the ICSI operation.

The mTESE surgical approach was similar to that described by authors Jensen C et al. [6]. The surgeon used a scalpel to make a transverse incision in the anterior middle of the scrotum, extruded the testis, and incised the meatus and the following tissues to reveal the testicular leucorrhaphy. The testis was fixed with the left hand. A 1-cm transverse incision was made in the white membrane of the anterior middle of the testis under 6x surgical magnification to reveal the testicular tissue. The blood vessels were electrocauterized to stop bleeding. The thick white varicocele was searched for under a 25x surgical microscope. The tubules that may contain spermatozoa were removed with ophthalmic scissors, and placed under a microscope to examine and search for spermatozoa. After obtaining sufficient spermatozoa, the wound was closed with adequate hemostasis.

### Controlled ovarian stimulation (COS), ICSI process, and definition of the label

The COS and ICSI operating procedures were similar to those described by authors Bedenk J et al. [19]. One or two blastocyst-stage embryos were transferred with luteal support per cycle. Serum human chorionic gonadotropin (HCG) ≥ 15 IU/L measured on the 14th day after transfer was considered positive for HCG, and the detection of a gestational sac on ultrasound on the 30th day after transfer was considered positive for clinical pregnancy.

### Feature engineering

Based on previous studies [20, 21] and expert opinions (3 independent specialists in andrology and reproductive medicine from the Second Affiliated Hospital of Wenzhou Medical University), we developed an initial predictive clinical pregnancy model with 22 variables as candidate independent variables (Supplementary Material 1). To eliminate the multicollinearity of the data and remove redundant features, we processed the data using Recursive Feature Elimination (RFE) to remove 1 feature (HCG) [22]. We used the ML-based Random Forest (RF) algorithm (missForest R package) to interpolate features with missing values less than 10% [23]. The models included in the final prediction of clinical pregnancy included 21 features, and 1 label, with 4 categorical variables and 17 continuous variables. We use the MinMaxScaler to normalize the data for continuous features and a one-hot code for categorical features [24]. There is no significant data imbalance in the labeling of this study therefore no Synthetic Minority Oversampling Technique (SMOTE) processing of the data was required [25]. We randomly split all the data in a ratio of 80:20, where 80% is used as a training set to train the models and 20% is used as a test set to test the models.

### Predictive modeling strategy

We used six ML algorithms, including k-nearest neighbor (KNN), support vector machine (SVM), RF, categorical boosting (CatBoost), extreme gradient boosting (XGBoost), and gradient boosting decision tree (GBDT) to develop the prediction models. We used a grid search to tune the hyperparameters and 5-fold cross-validation to obtain the optimal combination of hyperparameters for optimal model performance in predicting clinical pregnancy. Different ML algorithms have their data-applicable characteristics, so we trained six different ML models to predict clinical pregnancies to test the reliability, accuracy, and robustness of the models. We evaluated the performance and robustness of the predictive models by calculating the area under the receiver operating characteristic curve (AUROC), accuracy, precision, recall, F1 score, brier score, and the area under the precision-recall (P-R) curve (AP) for each predictive clinical pregnancy ML model. After comparing the performance discriminant characteristics of each ML model for predicting clinical pregnancy, the model with the best AUROC performance was selected as the optimal model for predicting clinical pregnancy, and the decision-making process of the model was interpreted using the SHAP.

### Interpretation of the model using the SHAP

The SHAP generates a SHAP value for each feature of the ML model to determine the value of the feature’s contribution to the clinical pregnancy prediction, with a positive or negative SHAP value indicating a positive or negative influence on the feature’s contribution to the clinical pregnancy prediction [26]. The SHAP summary plot provides a direct view of the importance of each feature and the contribution of each feature to the output of the ML model, while the SHAP force plot provides a visual understanding of how the ML model makes decisions about clinical pregnancy prediction [27].

### Statistical analysis

Continuous variables with normal distribution were expressed as mean ± standard deviation (SD) according to the data distribution; otherwise, they were expressed as median and interquartile range, and comparisons between groups were made using the Mann-Whitney U test. Categorical variables were expressed as frequencies (percentages) using Pearson’s Chi-square test or Fisher’s exact test, and a two-sided *P* < 0.05 was considered statistically significant. Statistical analysis of data between groups was performed using R 4.3.1. The R packages used were tidyverse, haven, gtsummary, MASS, missForest, and caret. ML models were analyzed using Python 3.11 software using scikit- learn1.3.0.

## Results

### Baseline characteristics of participants

**Fig. 1:**
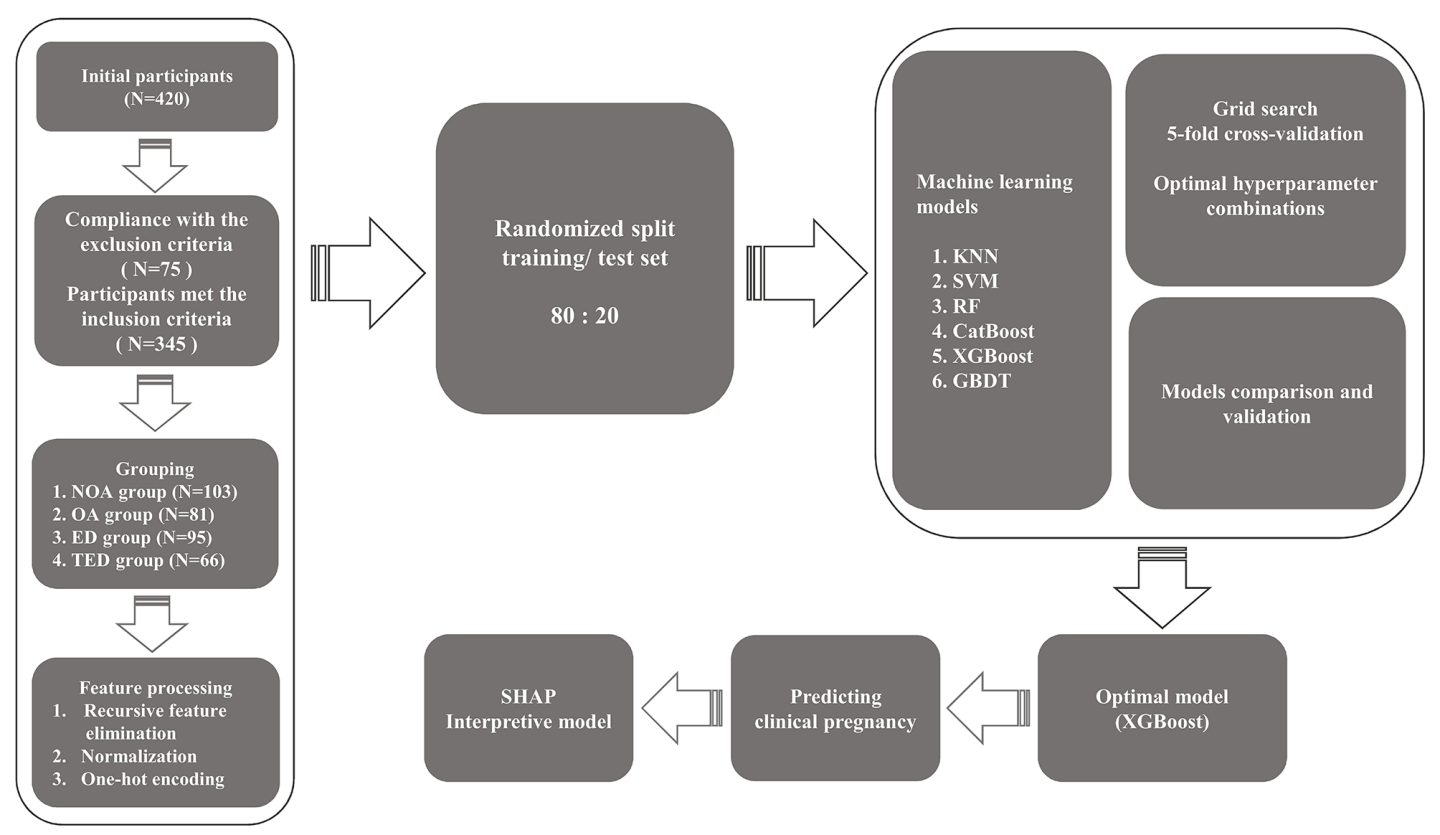
Participant screening and study design flowchart

Figure 1 illustrates the participant screening and study design process for this study. Table 1 summarizes the baseline characteristics of the participants. Age, body mass index (BMI), tobacco use, FSH, Irisin, Nesfatin-1, NOA group, and ED group were significantly lower in the male participants in the clinical pregnancy group than in the non-clinical pregnancy group, with statistically significant differences between groups (*P* < 0.05). In contrast, the testicular volume (TV), total testosterone (TT), Inhibin B, Johnsen score, and TED groups of male participants in the clinical pregnancy group were significantly higher than those in the non-clinical pregnancy group, with statistically significant differences between groups (*P* < 0.05). The BMI, Age, and FSH of the female participants in the clinical pregnancy group were significantly lower than those in the non-clinical pregnancy group, and there was a statistically significant difference between the groups (*P* < 0.05). In contrast, the antral follicle count (AFC) and AMH of female participants in the clinical pregnancy group were significantly higher than those in the non-clinical pregnancy group, with statistically significant differences between groups (*P* < 0.05). In addition, we separately counted the baseline characteristics of the OA, NOA, and TED groups. In the NOA group, the males had a TV (ml): 10 (9, 11), FSH (IU/L): 15.07 (12.19, 16.01), TT (ng/ml): 4.41 (4.09, 4.94), and Johnsen score: 8 (8, 8). In the OA group, the males had a TV (ml): 12 (10, 12), FSH (IU/L): 13.13 (11.72, 15.01), TT (ng/ml): 4.67 (4.15, 5.07), Johnsen score: 10 (10, 10). The results of pre-cycle semen analysis in the TED group were sperm concentration (10^6^/ml): 50.95 (34.67, 70.46); sperm progressive motility (%): 50.80 (41.33, 61.17), sperm total motility (%): 67.81 (58.33, 78.17), sperm normal forms (%): 5.16 (4.73, 6.23), sperm DNA fragmentation index (%): 15.21 (11.18, 19.57).

**Table 1:** Baseline characteristics of participants

Characteristic	No clinical pregnancy (*N* = 192)	Clinical pregnancy (*N* = 153)	*P*-value
**Male**			
Age (years)	37 (34, 39)	35 (31, 38)	< 0.001 ^a^
BMI (kg/m^2^)	26.18 (24.86, 26.84)	24.86 (24.20, 25.52)	< 0.001 ^a^
Tobacco use			< 0.001 ^b^
No	63 (32.81%)	116 (75.82%)	
Yes	129 (67.19%)	37 (24.18%)	
TV (ml)	10 (10, 12)	12 (11, 13)	< 0.001 ^a^
FSH (IU/L)	14.31 (12.66, 15.48)	12.19 (10.66, 13.60)	< 0.001 ^a^
LH (IU/L)	6.13 (4.60, 7.47)	5.90 (4.84, 7.34)	0.700 ^a^
E2 (pg/ml)	37.4 (33.4, 41.9)	38.1 (33.8, 43.0)	0.200 ^a^
TT (ng/ml)	4.28 (4.02, 4.81)	4.81 (4.54, 5.07)	< 0.001 ^a^
Inhibin B (pg/ml)	76 (72, 86)	86 (81, 90)	< 0.001 ^a^
Irisin (ng/ml)	162.36 (159.72, 163.67)	159.72 (158.41, 161.04)	< 0.001 ^a^
Nesfatin-1 (ng/ml)	5.36 (5.09, 5.57)	5.09 (4.95, 5.29)	< 0.001 ^a^
TED group			< 0.001 ^b^
No	183 (95.31%)	96 (62.75%)	
Yes	9 (4.69%)	57 (37.25%)	
NOA group			< 0.001 ^b^
No	116 (60.42%)	126 (82.35%)	
Yes	76 (39.58%)	27 (17.65%)	
ED group			0.027 ^b^
No	130 (67.71%)	120 (78.43%)	
Yes	62 (32.29%)	33 (21.57%)	
OA group			> 0.900 ^b^
No	147 (76.56%)	117 (76.47%)	
Yes	45 (23.44%)	36 (23.53%)	
Johnsen score	9 (8, 9)	10 (9, 10)	< 0.001 ^a^
**Female**			
BMI (kg/m2)	25.92 (23.04, 27.36)	23.04 (21.60, 24.48)	< 0.001 ^a^
Age (years)	36 (32, 38)	30 (28, 33)	< 0.001 ^a^
AFC (n)	9 (7, 13)	13 (11, 15)	< 0.001 ^a^
AMH (ng/ml)	2.70 (2.10, 3.70)	3.90 (3.30, 4.50)	< 0.001 ^a^
FSH (IU/L)	11.58 (10.29, 12.22)	9.97 (8.68, 10.93)	< 0.001 ^a^
HCG			< 0.001 ^b^
Negative	168 (87.50%)	0 (0.00%)	
Positive	24 (12.50%)	153 (100.00%)	

^a^ Mann-Whitney U test, ^b^ Pearson’s Chi-square test, or Fisher’s exact test

### Performance comparison of ML models for predicting clinical pregnancy

**Fig. 2:**
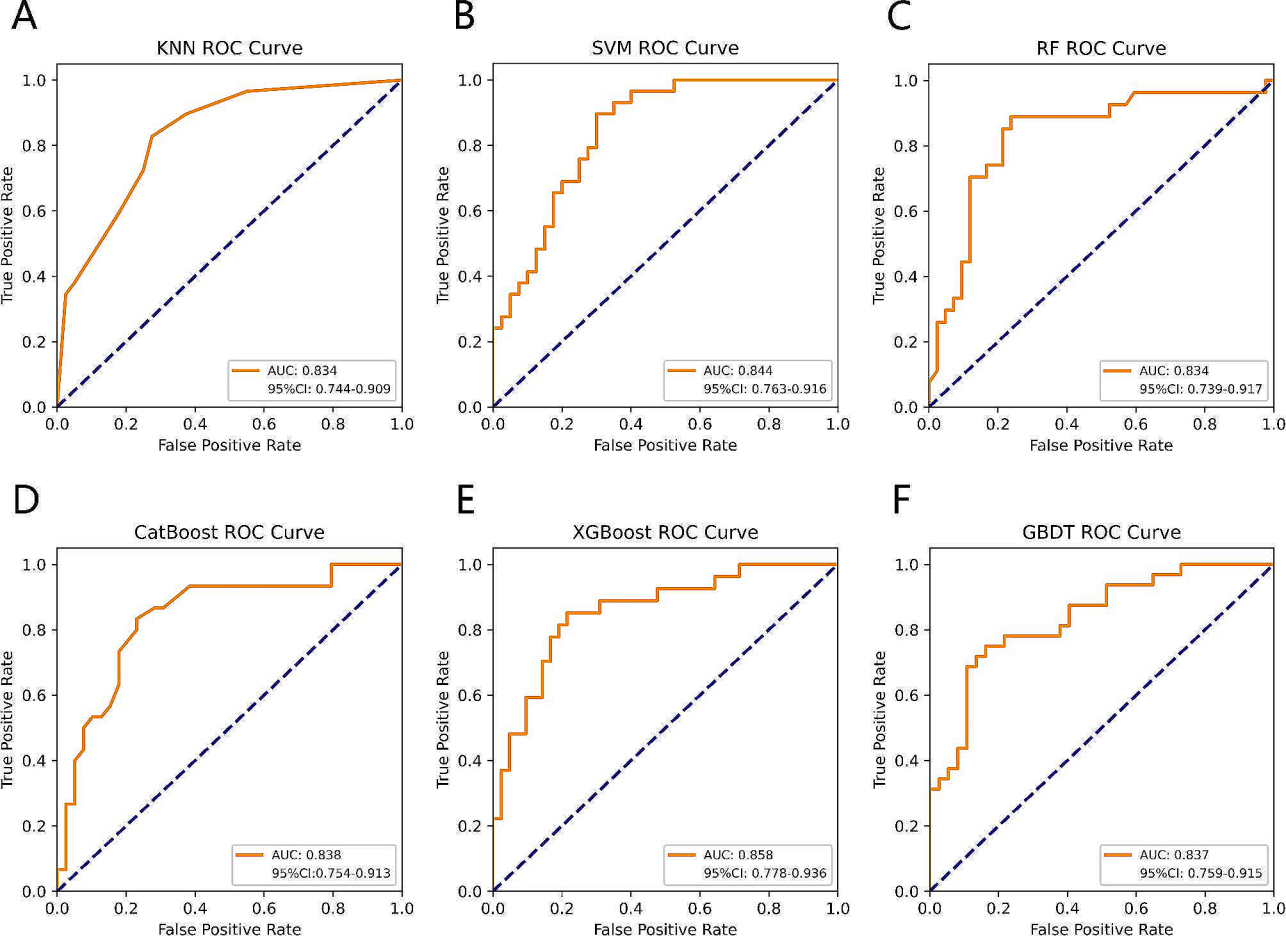
AUROC comparison of six ML models for predicting clinical pregnancy. (**A**) KNN; (**B**) SVM; (**C**) RF; (**D**) CatBoost; (**E**) XGBoost; (**F**) GBDT

**Fig. 3:**
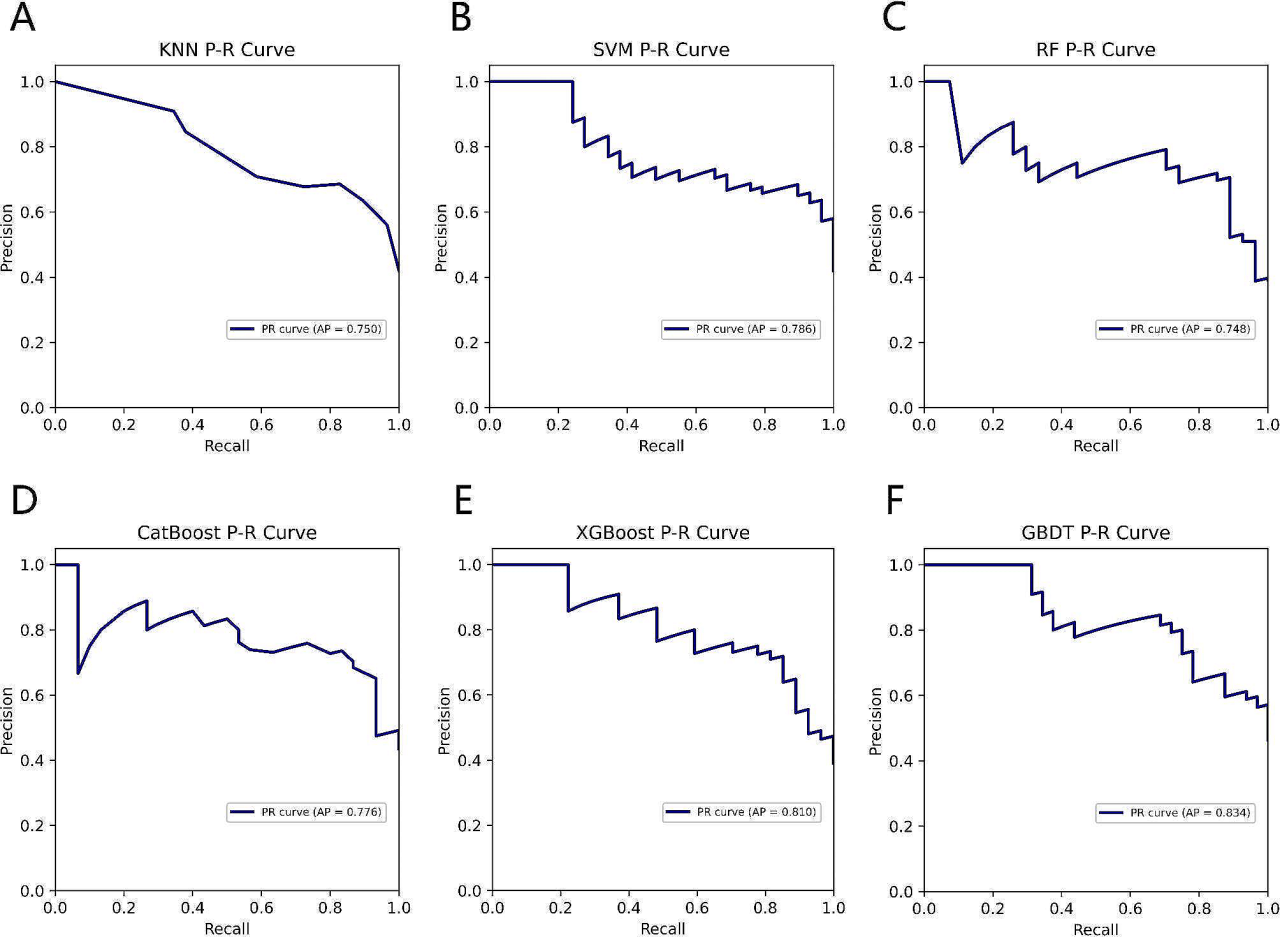
Comparison of P-R curves of six ML models for predicting clinical pregnancy. (**A**) KNN; (**B**) SVM; (**C**) RF; (**D**) CatBoost; (**E**) XGBoost; (**F**) GBDT

**Fig. 4:**
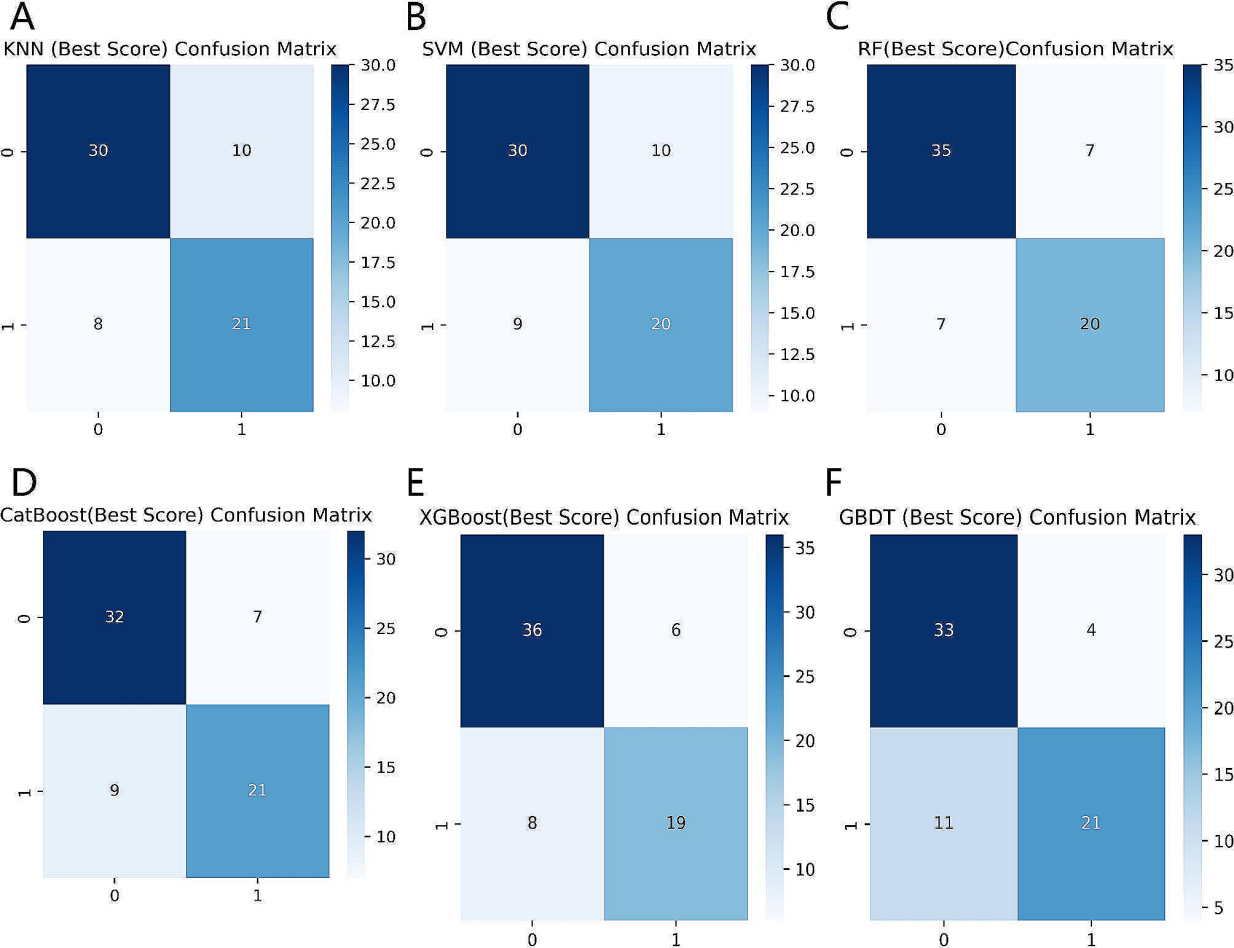
Confusion matrix comparison of six ML models for predicting clinical pregnancy. (**A**) KNN; (**B**) SVM; (**C**) RF; (**D**) CatBoost; (**E**) XGBoost; (**F**) GBDT

Table 2 summarizes the performance of the six ML models in predicting clinical pregnancy. Among them, the XGBoost model best predicted clinical pregnancy with AUROC of 0.858, 95% confidence interval (CI) of 0.778–0.936, and AP of 0.810 (Figs. 2 and 3). The accuracy of the XGBoost model was 79.71% with the F1 score of 0.731, and a comprehensive analysis based on the performance of the six ML models showed that the XGBoost had the highest accuracy and robustness in predicting clinical pregnancy (Fig. 4).

**Table 2:** Performance evaluation of six machine learning models

Characteristics	KNN	SVM	RF	CatBoost	XGBoost	GBDT
AUROC	0.834	0.844	0.834	0.838	0.858	0.837
95% CI	0.744–0.909	0.763–0.916	0.739–0.917	0.754–0.913	0.778–0.936	0.759–0.915
Accuracy	73.91%	72.46%	79.71%	76.81%	79.71%	78.26%
Precision	67.74%	66.67%	74.07%	75.00%	76.00%	84.00%
Recall	72.41%	68.97%	74.07%	70.00%	70.37%	65.63%
F1 score	0.700	0.678	0.741	0.724	0.731	0.737
Brier score	0.169	0.178	0.155	0.160	0.151	0.177
AP	0.750	0.786	0.748	0.776	0.810	0.834

### The XGBoost model SHAP features importance and individual decision-making

**Fig. 5:**
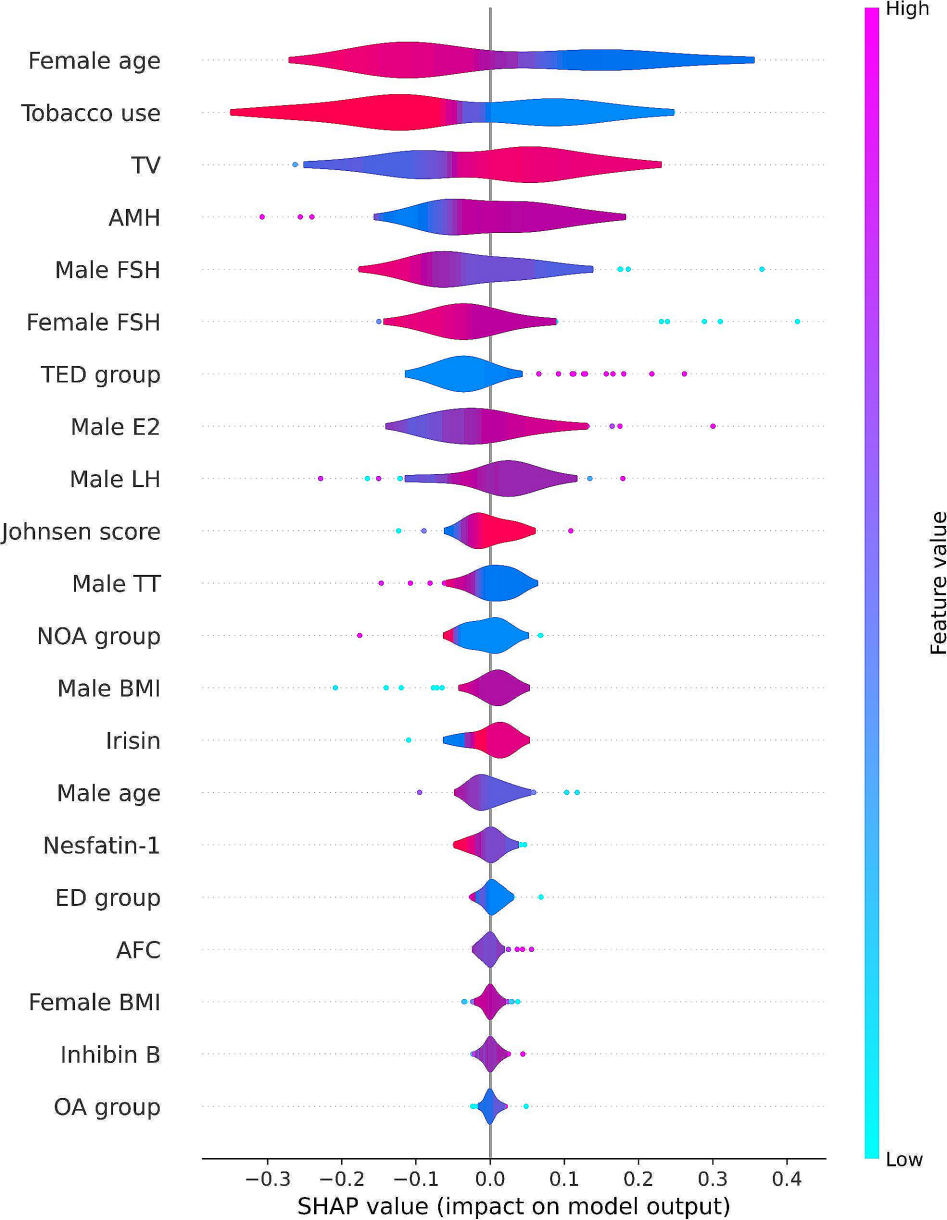
The SHAP global summary plot of the XGBoost model predicting clinical pregnancy

**Fig. 6:**
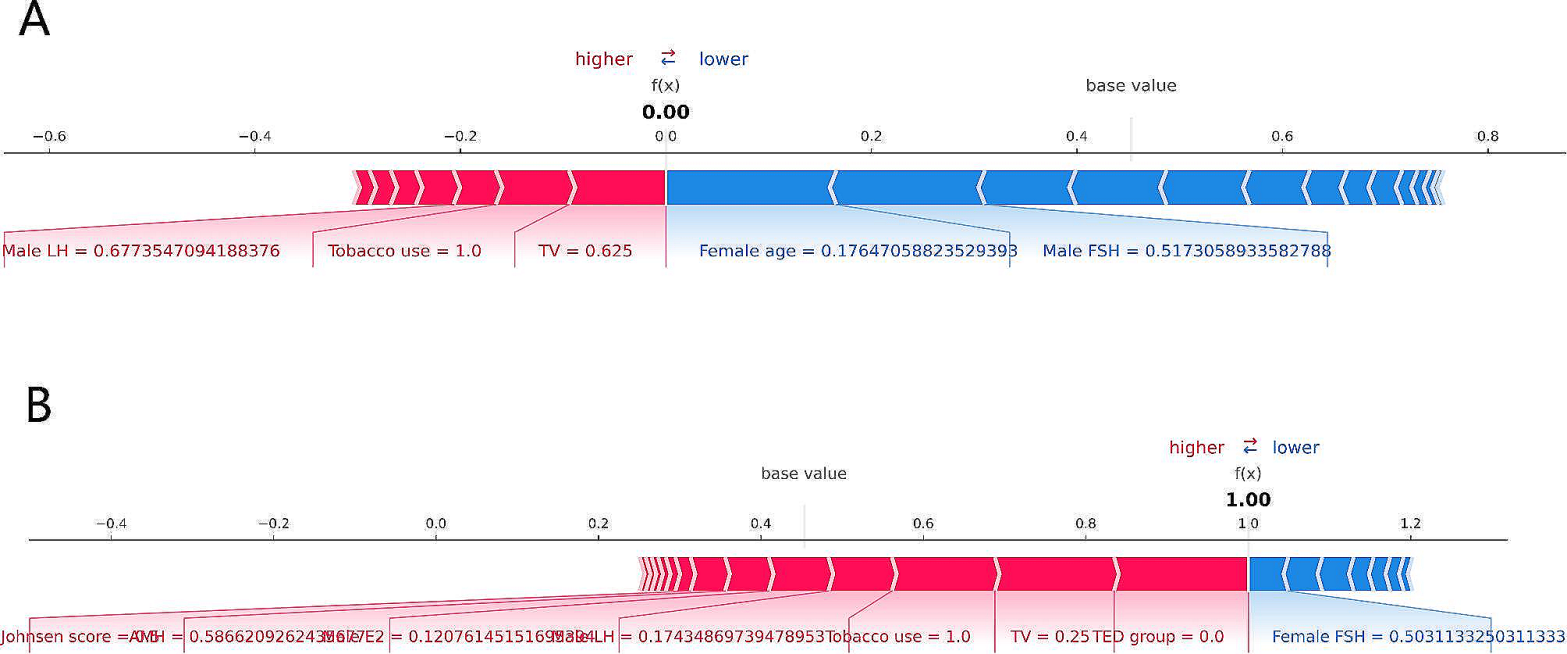
The SHAP force plots for different prediction outcomes. (**A**) non-clinical pregnancy, (**B**) clinical pregnancy

We used SHAP global summary plots to visualize the effect of each feature in the XGBoost model on the prediction of clinical pregnancy in the test dataset. The importance of the SHAP plot features suggests that the age of the female is the most important feature in the XGBoost model for predicting clinical pregnancy. The SHAP plot showed that younger age of women, higher TV, non-tobacco use, higher AMH, lower FSH in females, lower FSH in males, the TED group, and the non-NOA group all resulted in an increased probability of clinical pregnancy (Fig. 5). Figure 6 illustrates individual force plot for (A) non-clinical pregnancy and (B) clinical pregnancy. The horizontal axis in the force plot represents the predicted value of clinical pregnancy probability f(x) and is labeled with the base value, and the SHAP values labeled below indicate the contribution of each feature in the XGBoost model to the prediction of clinical pregnancy. Red arrows on the left side indicate features that increase the probability of clinical pregnancy, blue arrows on the right side indicate features that decrease the probability of clinical pregnancy, and the length of the arrows indicates the degree to which each feature contributes to the prediction of clinical pregnancy. The red and blue intersections represent individual predictions from the XGBoost model, with 0 indicating a non-clinical pregnancy and 1 indicating a clinical pregnancy.

## Discussion

We successfully trained and tested six ML predictive models for clinical pregnancy in ICSI with surgical testicular sperm retrieval for different etiologies. We found that the XGBoost model performed the best and was selected for predicting clinical pregnancies. The average AUROC of the XGBoost model in the test data was 0.858 (95% CI: 0.778–0.936), representing excellent model efficiency and robustness. We used SHAP global summary plots to show the importance of each feature in the XGBoost model, suggesting that female age is the most important feature. The probability of clinical pregnancy was higher in patients with younger female age, bigger TV, non-tobacco use, higher AMH, lower FSH in females, lower FSH in males, TED, and non-NOA. Our SHAP force plot clearly shows how the XGBoost model makes individual decisions about non-clinical or clinical pregnancy and how each feature contributes to the predicted outcome, allowing the users to trust and understand the model more. To the best of our knowledge, this is the first study to apply ML methods to predict clinical pregnancy in ICSI using testicular spermatozoa of different etiologies. The use of testicular sperm for ICSI has become the mainstay of treatment at IVF centers for infertile couples who are unable to provide their semen and a sufficient amount of competent sperm on the day of oocyte retrieval. Not all infertile couples are successful in achieving a clinical pregnancy with this treatment option. For this reason, there is an urgent need for infertile couples to understand the possible clinical pregnancy outcomes of ICSI using testicular sperm to inform their weighing of acceptable treatment risks and costs. Therefore, the ML clinical pregnancy prediction model we developed using testicular spermatozoa for different etiologies has important clinical applications.

Song J et al. used different types of acquired, idiopathic, and congenital azoospermia for predicting clinical pregnancy outcomes [28]. They constructed a logistic regression prediction model, which showed that bigger TV, higher testosterone levels, younger age of the woman, bigger AFC, and higher AMH were associated with a higher probability of clinical pregnancy. This is consistent with our findings suggesting that better ovarian and testicular function are associated with successful clinical pregnancy. We used an interpretable ML approach to construct a clinical pregnancy prediction model with more powerful data analysis capabilities than traditional logistic regression and effectively improved the accuracy and robustness of predicting clinical pregnancy. The SHAP summary plot we plotted suggests that female age is the most important model feature. Similar to our findings, Kato K et al. constructed an IVF clinical pregnancy prediction model using only female age and embryo developmental rate as independent variables, and their study showed that female age has a strong relationship with IVF clinical pregnancy [29]. Tsafrir A et al. studied clinical pregnancy in IVF using frozen oocytes and showed a significant negative correlation between female age and oocyte quality [30]. This suggests that female age affects the final clinical pregnancy outcome by influencing oocyte quality. Li F et al. studied and constructed a clinical pregnancy prediction model for females with poor ovarian response to IVF and ICSI treatment, suggesting that women older than 35 years, with higher BMI, and higher basal FSH were associated with lower clinical pregnancy rates [31]. This is in general consistent with our findings suggesting that older female age, higher BMI, and higher basal FSH are negatively associated with clinical pregnancy.

In addition to female factors being associated with clinical pregnancy outcomes, male factors are also strongly implicated. Our findings showed that the TED group had a higher clinical pregnancy rate than the other groups, while the NOA group had the lowest clinical pregnancy rate, and we hypothesized that it might be related to the fact that the TED group had higher sperm quality, while the NOA group had poorer sperm quality. Our testicular histopathologic analysis also confirmed that the NOA group had a lower Johnsen score than the other groups. Similar conclusions were reached by Aboukhshaba A et al. who concluded that the fertilization and live birth rates of NOA testicular spermatozoa obtained by m-TESE were relatively low [32]. We counted the sperm parameters of the TED group before the ICSI cycle and showed that sperm concentration, progressive motility, sperm normal forms, and sperm DNA fragmentation index were within the normal reference ranges, which represented a high sperm quality in the TED group. Our data showed that the ratio of TED patients to total ICSI cycles was 19.13%, which may be related to the influence of lifestyle habits, the environment in which spermatozoa are obtained, and multiple psychological factors. Zhang X et al. showed that nearly 10% of males in the total cycle of IVF and ICSI had TED, which was related to the fact that males undergoing IVF treatment were often worried about abnormal semen results, the outcome of IVF treatment, socio-economic pressures, and psychiatric disorders such as anxiety and depression, which were very common among males undergoing IVF treatment [33]. Similar views were expressed by Wang J et al., who investigated and found that the prevalence of male TED on the day of female ovum acquisition was 8.3% [34]. According to epidemiologic surveys, up to 52% of diabetic men experience ED, and 35–50% have ejaculatory disorders. We hypothesized that the effect of the ED group on clinical pregnancy may be related to disturbances in glucose metabolism, which may affect sperm quality [35, 36]. Service CA et al. made a similar point, suggesting that male obesity, diabetes, and metabolic syndrome negatively affect all sperm parameters and that male obesity, diabetes, and metabolic syndrome negatively correlate with clinical pregnancy and live birth rates, whether conceived through natural fertilization or assisted reproductive technology [37].

We trained and tested six ML models and chose the XGBoost model as the optimal model to predict clinical pregnancies associated with surgical sperm retrieval of different etiologies, which has several strengths and limitations. First, we used the SHAP to interpret the XGBoost model for predicting clinical pregnancy, making it easier for users to understand the model’s decision-making process and trust the model. Second, we utilize the powerful data analysis capabilities of ML to construct clinical pregnancy prediction models with higher efficiency, accuracy, and robustness than traditional models. Third, the clinical pregnancy prediction model we developed for using testicular spermatozoa with different etiologies can provide valuable clinical counseling strategies for such infertile couples. Fourth, our study also has the limitation that the amount of data collected in this study needs to be further improved, which is determined by the prevalence of the disease.

## Conclusions

Our developed XGBoost model for predicting clinical pregnancy based on surgical retrieval of testicular spermatozoa from different etiologies has high accuracy, efficiency, and robustness. Our use of the SHAP makes the XGBoost model more interpretable and can provide accurate, efficient, and practical clinical counseling decisions for such infertile couples. We will further extend the model features and update the training and validation data of the model to generalize the applicability of this clinical pregnancy prediction model to IVF centers in different countries and further improve the interpretability of the XGBoost model.

### Electronic supplementary material

Below is the link to the electronic supplementary material.

Supplementary Material 1

## Data Availability

The datasets used and/or analyzed during the current study are available from the corresponding author upon reasonable request.

## Abbreviations

ICSI: Intracytoplasmic sperm injection
OA: Obstructive azoospermia
NOA: Non-obstructive azoospermia
IVF: In vitro fertilization
TESA: Testicular sperm aspiration
mTESE: Microdissection testicular sperm extraction
ED: Erectile dysfunction
TED: Temporary ejaculatory disorders
DNA: Deoxyribonucleic acid
ML: Machine learning
SHAP: SHapley Additive exPlanation
IIEF-5: International index of erectile function 5
COS: Controlled ovarian stimulation
HCG: Human chorionic gonadotropin
RFE: Recursive Feature Elimination
SMOTE: Synthetic Minority Oversampling Technique
KNN: K-nearest neighbor
SVM: Support vector machine
CatBoost: Categorical boosting
XGBoost: Extreme gradient boosting
GBDT: Granding boosting decision tree
RF: Random Forest
AUROC: Area under the receiver operating characteristic curve
AP: Area under the P-R curve
FSH: Follicle-stimulating hormone
BMI: Body mass index
TV: Testicular volume
TT: Total testosterone
AFC: Antral follicle count
AMH: Anti-Müllerian hormone
SD: Standard deviation
IQR: Interquartile
CI: Confidence interval

## References

[1] Agarwal A, Baskaran S, Parekh N, et al. Male Infertility Lancet. 2021;397(10271):319–33. 33308486 10.1016/S0140-6736(20)32667-2

[2] Chen H, Wang C, Zhou H, et al. Laser-assisted selection of immotile spermatozoa has no effect on obstetric and neonatal outcomes of TESA-ICSI pregnancies. Reprod Biol Endocrinol. 2021;19(1):159. 34641894 10.1186/s12958-021-00835-9 PMC8507098

[3] Eisenberg ML, Esteves SC, Lamb DJ, et al. Male infertility. Nat Rev Dis Primers. 2023;9(1):49. 37709866 10.1038/s41572-023-00459-w

[4] Sharma A, Minhas S, Dhillo WS, Jayasena CN. Male infertility due to testicular disorders. J Clin Endocrinol Metab. 2021;106(2):e442–442459. 33295608 10.1210/clinem/dgaa781 PMC7823320

[5] Chen Z, Zhang D, Zhen J, Sun Z, Yu Q. Predicting cumulative live birth rate for patients undergoing in vitro fertilization (IVF)/intracytoplasmic sperm injection (ICSI) for tubal and male infertility: a machine learning approach using XGBoost. Chin Med J (Engl). 2022;135(8):997–9. 35730375 10.1097/CM9.0000000000001874 PMC9276286

[6] Jensen C, Ohl DA, Fode M, et al. Microdissection testicular sperm extraction Versus multiple needle-pass percutaneous testicular sperm aspiration in men with nonobstructive azoospermia: a Randomized Clinical Trial. Eur Urol. 2022;82(4):377–84. 35599183 10.1016/j.eururo.2022.04.030

[7] Levi-Setti PE, Negri L, Baggiani A, et al. Testicular sperm extraction and intracytoplasmic sperm injection outcome in cancer survivors with no available cryopreserved sperm. J Assist Reprod Genet. 2020;37(4):875–82. 31981037 10.1007/s10815-020-01697-7 PMC7183024

[8] Liu X, Gao M, Sun J, et al. Effects of testicular sperm aspiration upon first cycle ICSI-ET for type 2 diabetic male patients. Syst Biol Reprod Med. 2020;66(6):355–63. 32717167 10.1080/19396368.2020.1785042

[9] Sakkas D, Alvarez JG. Sperm DNA fragmentation: mechanisms of origin, impact on reproductive outcome, and analysis. Fertil Steril. 2010;93(4):1027–36. 20080235 10.1016/j.fertnstert.2009.10.046

[10] Peiffer-Smadja N, Rawson TM, Ahmad R, et al. Machine learning for clinical decision support in infectious diseases: a narrative review of current applications. Clin Microbiol Infect. 2020;26(5):584–95. 31539636 10.1016/j.cmi.2019.09.009

[11] VerMilyea M, Hall J, Diakiw SM, et al. Development of an artificial intelligence-based assessment model for prediction of embryo viability using static images captured by optical light microscopy during IVF. Hum Reprod. 2020;35(4):770–84. 32240301 10.1093/humrep/deaa013 PMC7192535

[12] Cooray SD, Boyle JA, Soldatos G, et al. Protocol for development and validation of a clinical prediction model for adverse pregnancy outcomes in women with gestational diabetes. BMJ Open. 2020;10(11):e038845. 33154055 10.1136/bmjopen-2020-038845 PMC7646337

[13] Valiente Fernández M, Lesmes González de Aledo A, Delgado Moya FP. Martín Badía I. SHAP model explainability in ECMO-PAL mortality prediction: a critical analysis. Intensive Care Med. 2023;49(12):1559. 37906260 10.1007/s00134-023-07252-z

[14] Salonia A, Bettocchi C, Boeri L, et al. European Association of Urology Guidelines on sexual and Reproductive Health-2021 update: male sexual dysfunction. Eur Urol. 2021;80(3):333–57. 34183196 10.1016/j.eururo.2021.06.007

[15] Mazzilli F. Erectile Dysfunction: causes, diagnosis and treatment: an update. J Clin Med. 2022;11(21):6429. 36362656 10.3390/jcm11216429 PMC9657711

[16] Mazzilli R, Vaiarelli A, Dovere L, et al. Severe male factor in in vitro fertilization: definition, prevalence, and treatment. An update. Asian J Androl. 2022;24(2):125–34. 34259196 10.4103/aja.aja_53_21 PMC8887096

[17] Brant A, Schlegel PN. Modern surgical treatment of azoospermia. Curr Opin Urol. 2023;33(1):39–44. 36301052 10.1097/MOU.0000000000001055

[18] Cito G, Coccia ME, Sessa F, et al. Testicular fine-needle aspiration for sperm Retrieval in Azoospermia: a small step toward the Technical Standardization. World J Mens Health. 2019;37(1):55–67. 30584991 10.5534/wjmh.180077 PMC6305866

[19] Bedenk J, Režen T, Železnik Ramuta T, et al. Recombinant anti-Müllerian hormone in the maturation medium improves the in vitro maturation of human immature (GV) oocytes after controlled ovarian hormonal stimulation. Reprod Biol Endocrinol. 2022;20(1):18. 35073905 10.1186/s12958-022-00895-5 PMC8785574

[20] Zhang HL, Mao JM, Liu DF, et al. Clinical outcomes of microdissection testicular sperm extraction-intracytoplasmic sperm injection with fresh or cryopreserved sperm in patients with nonobstructive azoospermia. Asian J Androl. 2021;23(2):211–4. 32719193 10.4103/aja.aja_38_20 PMC7991819

[21] Aydos K, Aydos OS. Sperm selection procedures for optimizing the outcome of ICSI in patients with NOA. J Clin Med. 2021;10(12):2687. 34207121 10.3390/jcm10122687 PMC8234729

[22] Deng F, Zhao L, Yu N, Lin Y, Zhang L. Union with recursive feature elimination: a feature selection Framework to improve the classification performance of Multicategory causes of Death in Colorectal Cancer. Lab Invest. 2023;104(3):100320. 38158124 10.1016/j.labinv.2023.100320

[23] Chen X, Chen H, Nan S, Kong X, Duan H, Zhu H. Dealing with missing, Imbalanced, and sparse features during the development of a Prediction Model for Sudden Death using Emergency Medicine Data: Machine Learning Approach. JMIR Med Inf. 2023;11:e38590. 10.2196/38590 PMC9898833 36662548

[24] Ren Y, Chakraborty T, Doijad S, et al. Prediction of antimicrobial resistance based on whole-genome sequencing and machine learning. Bioinformatics. 2022;38(2):325–34. 34613360 10.1093/bioinformatics/btab681 PMC8722762

[25] Dablain D, Krawczyk B, Chawla NV. DeepSMOTE: Fusing Deep Learning and SMOTE for Imbalanced Data. IEEE Trans Neural Netw Learn Syst. 2023;34(9):6390–404. 35085094 10.1109/TNNLS.2021.3136503

[26] Cao S, Hu Y. Interpretable machine learning framework to predict gout associated with dietary fiber and triglyceride-glucose index. Nutr Metab (Lond). 2024;21(1):25. 38745171 10.1186/s12986-024-00802-2 PMC11092237

[27] Cao S, Hu Y. Creating machine learning models that interpretably link systemic inflammatory index, sex steroid hormones, and dietary antioxidants to identify gout using the SHAP (SHapley Additive exPlanations) method. Front Immunol. 2024;15:1367340. 38751428 10.3389/fimmu.2024.1367340 PMC11094226

[28] Song J, Gu L, Ren X, et al. Prediction model for clinical pregnancy for ICSI after surgical sperm retrieval in different types of azoospermia. Hum Reprod. 2020;35(9):1972–82. 32730569 10.1093/humrep/deaa163

[29] Kato K, Ueno S, Yabuuchi A, et al. Women’s age and embryo developmental speed accurately predict clinical pregnancy after single vitrified-warmed blastocyst transfer. Reprod Biomed Online. 2014;29(4):411–6. 25129691 10.1016/j.rbmo.2014.06.007

[30] Tsafrir A, Ben-Ami I, Eldar-Geva T, et al. Clinical outcome of planned oocyte cryopreservation at advanced age. J Assist Reprod Genet. 2022;39(11):2625–33. 36264444 10.1007/s10815-022-02633-7 PMC9723084

[31] Li F, Lu R, Zeng C, Li X, Xue Q. Development and validation of a clinical pregnancy failure prediction model for poor ovarian responders during IVF/ICSI. Front Endocrinol (Lausanne). 2021;12:717288. 34497586 10.3389/fendo.2021.717288 PMC8419272

[32] Aboukhshaba A, Punjani N, Doukakis S, Zaninovic N, Palermo G, Schlegel PN. Testicular sperm characteristics in men with nonobstructive azoospermia and their impact on intracytoplasmic sperm injection outcome. Fertil Steril. 2022;117(3):522–7. 34674828 10.1016/j.fertnstert.2021.09.024

[33] Zhang X, Yang L, Wang W, Yang L. Psychological distress, emotion regulation, neuroticism, and sexual relationship on patients with temporary ejaculation failure in vitro fertilization-embryo transfer treatment. Front Psychol. 2022;13:1090244. 36687954 10.3389/fpsyg.2022.1090244 PMC9853009

[34] Wang J, Xue M. Influence of age, stigma and social support on male temporary ejaculation failure on IVF oocyte retrieval day. Reprod Biol Endocrinol. 2021;19(1):9. 33441151 10.1186/s12958-020-00691-z PMC7805061

[35] Laumann EO, Paik A, Rosen RC. Sexual dysfunction in the United States: prevalence and predictors. JAMA. 1999;281(6):537–44. 10022110 10.1001/jama.281.6.537

[36] Desai A, Chen R, Cayetano A, Jayasena CN, Minhas S. Understanding and treating ejaculatory dysfunction in men with diabetes mellitus. Andrology. 2023;11(2):379–98. 35933708 10.1111/andr.13262

[37] Service CA, Puri D, Al Azzawi S, Hsieh TC, Patel DP. The impact of obesity and metabolic health on male fertility: a systematic review. Fertil Steril. 2023;120(6):1098–111. 37839720 10.1016/j.fertnstert.2023.10.017

