# Supplementary material for: Interpretable machine learning models for predicting clinical pregnancies associated with surgical sperm retrieval from testes of different etiologies: a retrospective study
Source: BMC Urol. 2024 Jul 29;24:156. doi: 10.1186/s12894-024-01537-1 (PMC11285258; doi:10.1186/s12894-024-01537-1)
Supplement: Supplementary file 1 — Supplementary Material 1 [file 12894_2024_1537_MOESM1_ESM.docx]

The initial candidate variables include Male age, Male BMI, Tobacco use, TV, Male FSH, Male LH, Male E2, Male TT, Inhibin B, Irisin, Nesfatin-1, TED group, NOA group, ED group, OA group, Johnsen score, Female BMI, Female age, AFC, AMH, Female FSH, HCG.
